# ACKNOWLEDGEMENTS TO REVIEWERS

**DOI:** 10.2174/1570159X2314250805160736

**Published:** 2025-08-05

Bentham Science Publishers would like to thank and appreciate the co-operation from all reviewers for their constructive comments and feedback on the manuscripts submitted to “**Current Neuropharmacology**”. Their efforts have contributed greatly to the high quality and continuous growth of the journal. Given below is the list of reviewers who reviewed articles for the Journal during 2025:

Sawsan Hammad Abuhammad (UAE)

Daniela Adamo (Italy)

Umberto Aguglia (Italy)

Gustav Akk (USA)

Hayder Mutter K. Al-Kuraishy (Iraq)

Marika Alborghetti (Italy)

Gamze Yayla Altun (Turkiye)

Raül Andero (Spain)

George Anderson (UK)

Mohd Imran Ansari (Saudi Arabia)

F. Anwar (Saudi Arabia)

Mohammad Ali Arami (Iran)

Antonietta Arcella (Italy)

Bala Asis (India)

Marco Atzori (Mexico)

Marãa Rosa Avila-Costa (Mexico)

Xianshu Bai (Germany)

Manjeshwar Shrinath Baliga (India)

Santiago J. Ballaz (Ecuador)

Cindy Rodrãguez Bandala (Mexico)

Nagaraju Bandaru (India)

Maria Barbaccia (Italy)

Christian Barbato (Italy)

Colin J Barnstable (USA)

Agnieszka Basta-Kaim (Poland)

Anne Batisse (France)

Benan Bayrakci (Turkiye)

Roy G. Beran (Australia)

Shahnawaz Ali Bhat (India)

Arjan Blokland (Netherlands)

Robin E. Bonomi (USA)

Jonathan M. Borkum (USA)

David J. Brooks (Denmark)

David Brooks (UK)

Vittorio Calabrese (Italy)

Milena Cannella (Italy)

Zhongcheng Cao (China)

Xiang Cao (China)

Joã£o Paulo Capela (Portugal)

C.R. Carlini (Brazil)

Diego Centonze (Italy)

Adnan Cetin (Turkiye)

Ting Ting Chang (Taiwan)

Lijia Chang (China)

Lei Chang (China)

Indranath Chatterjee (UK)

Zhong Chen (China)

Fangfang Chen (China)

Santenna Chenchula (India)

Rita Chiaramonte (Italy)

Santina Chiechio (Italy)

Anatoliy A. Chumak (Ukraine)

Nobrega Claudia (Portugal)

A. Copani (Italy)

Cinzia Coppola (Italy)

Shanshan Cui (China)

Emanuele Dâ€™Amico (Italy)

Srijit Das (Oman)

Daniel Dautan (Sweden)

Domenico De Berardis (Italy)

Mohammad Amin Dehghani (Iran)

Liang Dong (China)

Ruiguo Dong (China)

Weixia Duan (China)

Hamed M. Elgendy (Qatar)

Massimiliano Esposito (Italy)

Majid Hassanpour Ezzati (UK)

Xinyi Fang (China)

Iliya A. Fedotov (Russia)

Peijian Feng (China)

Guijuan Feng (China)

Mengzhao Feng (China)

Marco Fiore (Italy)

Florian Freudenberg (Germany)

Xiaoqin Fu (China)

Hualin Fu (China)

Fabio Fumagalli (Italy)

Mine Sibel Gãrãn (Turkiye)

Weiqiang Gao (China)

Zhengxiang Gao (China)

Rupesh Kumar Gautam (India)

Maryam Ghasemi-Kasman (Iran)

Nikolay V. Goncharov (Russia)

Daji Guo (China)

Ashanul Haque (Saudi Arabia)

Yuto Hasegawa (USA)

Renhong He (China)

Ahmed Mohamed Nabil Helaly (Egypt)

Congli Hu (China)

Shukun Hu (China)

Bing Hu (China)

Yuan Huang (China)

Rong Huang (China)

Shalam Mohamed Hussain (Saudi Arabia)

Pãter Illãs (Germany)

Paola Imbrici (Italy)

Takuma Inagawa (Japan)

Hanish Singh Jayasingh Chellammal (Malaysia)

Bingyuan Ji (China)

Shujun Jiang (China)

Yantao Jin (China)

Wolfgang H. Jost (Germany)

Yiyuan Kang (China)

Devesh U. Kapoor (India)

Junichi Kitanaka (Japan)

Wojciech Kolodziejczyk (USA)

Jianglong Kong (China)

Marta Kot (Poland)

Georgios Kotzalidis (Italy)

Hongzhi Kuai (Japan)

Thomas Kuhn (USA)

Burak Kuzu (Turkiye)

Jacek Kwiecien (Canada)

Yulong Lan (China)

Yiran Lang (China)

D.A. Lanshakov (Russia)

Juan Le (China)

Si A. Lee (Korea, South)

Hongyi Lei (China)

Xingang Li (China)

Qinglin Li (China)

Lingling Li (China)

Ran Li (China)

Junjie Li (China)

Wen Li (China)

Jiada Li (China)

Meijuan Li (China)

Lifeng Li (China)

Mingfeng Li (China)

Zhenhu Liang (China)

Wei Sheng Lin (Taiwan)

Caixiu Lin (China)

Tomasz Litwin (Poland)

Xiaojin Liu (China)

Shuai Liu (China)

Ning Liu (China)

Chenyue Liu (China)

Qun Lu (China)

Xin Lu (China)

Di Luan (China)

Rong Ma (China)

Le Ma (China)

Mudasir Maqbool (India)

Victor P. Mathis (France)

Sidharth Mehan (India)

Jalil Mehrzad (Iran)

Jinfei Mei (China)

Seiko Miyata (Japan)

Hannah R. Monday (USA)

Ahmad Movahedpour (Iran)

Y. Muralimohanbabu (India)

Amirreza Naseri (Iran)

Alessandra Nicoletti (Italy)

Vladimir Nikolaevich Nikolenko (Russia)

Eva Maria Olivas-Flores (Mexico)

T. Dylan Olver (Canada)

Saad Omais (Lebanon)

Angelos Papadopoulos (Greece)

D. Pawlak (Poland)

Ke Pei (China)

Weifeng Peng (China)

Anastasia G. Peshkovskaya (Russia)

Bartåomiej Pochwat (Poland)

Francesco Pontieri (Italy)

William Z. Potter (USA)

Javad Poursamimi (Iran)

S. Preethi (India)

Shaogang Qu (China)

Boris B. Quednow (Switzerland)

Md Ataur Rahman (USA)

Omid Rajabzadeh (Iran)

Prasanna Ramani (India)

Siqiang Ren (China)

Rui Rodrigues (France)

Gaetano Santulli (USA)

Edward M. Sellers (Canada)

Ali Sepehrinezhad (Iran)

Santhosh Sethuramanujam (India)

Bechan Sharma (India)

Zi Sheng (China)

Yun Shi (China)

Yiwu Shi (China)

Dario Siniscalco (Italy)

Charalampos Skarlis (Greece)

Qiancheng Song (China)

Juexian Song (China)

Hongwen Song (China)

George B. Stefano (Czechia)

Mikhail Yu Stepanichev (Russia)

Maksim V. Storozhuk (Ukraine)

Lingyan Su (China)

Jinhao Sun (China)

Fangling Sun (China)

Zaoyi Sun (China)

Zhihua Sun (China)

Birce Dilge Taåÿkän (USA)

Pengwen Tan (Singapore)

Yong Tang (China)

Marijan Tepeå (Croatia)

Yuji Tomizawa (Japan)

Gonzalo E. Torres (USA)

Amit Kumar Tripathi (India)

Kadir Uludag (China)

Juliana Ferreira Vasques (Brazil)

Max Denisson Maurãcio Viana (Brazil)

Xiao Ping Wang (China)

Jue Wang (China)

Song Wang (China)

Xueqin Wang (China)

Aibo Wang (China)

Fei Wang (China)

Pengfei Wang (China)

Guihua Wei (China)

Zhisheng Wei (China)

Yanyan Wei (China)

Minghua Wu (China)

Yiyuan Xia (China)

Gelei Xiao (China)

Qian Xie (China)

Jianping Xie (China)

Biao Xu (China)

Jiaqing Yan (China)

Xiaoyu Yan (China)

Tianyi Yan (China)

Chuntao Yang (China)

Yong Yang (China)

Chenghao Yang (China)

Biying Yang (China)

Yong Yao (China)

Qing Ye (China)

Rwei-Ling Yu (Taiwan)

Lie Yu (China)

Bin Yu (China)

Wenbo Yu (China)

Xia Yue (China)

Nihal Yurteri (Turkiye)

Xianzhang Zeng (China)

Bi Zhang (China)

Yonggang Zhang (China)

Yuehui Zhang (China)

Ying Zhang (China)

Guangyu Zhang (China)

Hongcai Zhang (China)

Yongfang Zhang (China)

Ni Zhang (China)

Tong Zhao (China)

Zhenhua Zhao (China)

Haian Zheng (USA)

Xiaomin Zheng (China)

Ting Zheng (China)

Alexander V. Zholos (Ukraine)

Xiaopu Zhou (Hong Kong)

Xiaojuan Zhu (China)

Binglin Zhu (China)

Junyi Zhu (China)

Qianxing Zhuang (China)

Jie Zhuang (China)

Sergey M. Zimatkin (Belarus)

Zui Zou (China)

